# Linking variation in genome size to cyst size in chrysophytes

**DOI:** 10.1111/jpy.70217

**Published:** 2026-08-07

**Authors:** Natálie Protić, Iva Jadrná, Pavel Škaloud, Petr Knotek, Dora Čertnerová

**Affiliations:** ^1^ Institute of Microbiology of the Czech Academy of Sciences, Centre Algatech Třeboň Czech Republic; ^2^ Department of Botany, Faculty of Science Charles University Prague Czech Republic; ^3^ Research Department for Limnology University of Innsbruck Mondsee Austria

**Keywords:** chrysophytes, flow cytometry, genome size, *Mallomonas*, stomatocyst

## Abstract

Genome size varies tremendously across eukaryotes, which often contain far more DNA than expected from their biological complexity. To explain this paradox, selection‐based hypotheses propose that genome size evolves through selection acting on life‐history traits correlated with the phenotypic effects, independent of its genic content. To test the association between genome size and resting cyst size—a structure with morphology that may reflect overall body size—we selected chrysophyte algae (Chrysophyceae) producing siliceous stomatocysts as a model. In this study, we obtained and identified 85 chrysophyte strains representing 31 *Mallomonas* species using nuclear ITS rDNA region barcoding and estimated their genome sizes using propidium iodide flow cytometry. Within this genus, we observed more than a 75‐fold variation in genome size (0.15–11.25 pg) and frequent substantial intraspecific variation, in some cases consistent with whole‐genome doubling (polyploidization). By summarizing the published genome size records, adding our new measurements and combining them with published cyst sizes, we assembled a data set for 54 chrysophyte species to examine the relationship between genome size and cyst volume. Linear regression on log‐transformed data revealed a strong positive correlation ($R_{\mathrm{adj}}^{2}$ = 0.626, *p* < 0.001), showing that species with larger genomes tend to produce larger stomatocysts. In chrysophytes, this relationship is considerably stronger than the widely documented genome size–cell size correlation observed across eukaryotes. Our findings suggest that stomatocyst size is a more informative predictor of genome size in this group and highlight the potential for using stomatocyst sizes to infer ancestral genome sizes from the fossil record.

**Abbreviations**
BLAST: Basic Local Alignment Search Tool
CCD: charge‐coupled device
FCM: flow cytometry
G: gap
Gbp: giga base pair
ITS rDNA: internal transcribed spacer region of ribosomal DNA
Mbp: mega base pair
Na_2_HPO_4_
: disodium hydrogen phosphate
NCBI: National Center for Biotechnology Information
NCMA: The National Center for Marine Algae and Microbiota
nr: nuclear
PCR: polymerase chain reaction
pg: picogram
PI: propidium iodide
TEM: transmission electron microscopy
TES: N‐[Tris(hydroxymethyl)methyl]‐2‐aminoethanesulfonic acid
WC: Wright's Chu

## INTRODUCTION

Eukaryotic organisms exhibit tremendous variability in genome size, spanning more than five orders of magnitude (Gregory et al., 2007). While rapid advances in genomics have enabled detailed examination of how the coding DNA affects organism's phenotype, the consequences of the nuclear DNA amount itself on phenotype have received far less attention. However, genome size far exceeds what would be expected from differences in organisms' biological complexity, an observation termed the C‐value paradox (Thomas, 1971). Hypotheses explaining genome size variability operate with two main evolutionary drivers—the neutral and the adaptive. Although neutral processes such as mutation and genetic drift undoubtedly contribute to genome expansion or reduction, selection‐based theories have gained increasing attention, as genome size has shown to be correlated with numerous life‐history traits (Carta et al., 2022; Iannucci et al., 2022; Petrov, 2001; Yu et al., 2020; but see Morand & Ricklefs, 2005). Nuclear DNA content determines nucleus size (although some other mechanisms might be involved; Cantwell & Nurse, 2019), which also affects the cell size (Cavalier‐Smith, 2005). The genome size–cell size correlation, also known as the karyoplasmic ratio (Wilson, 1925), predicts that larger genomes are associated with lower metabolic rates or more slowly dividing cells, and these effects scale up to impact developmental rates, growth, and even body size (Cavalier‐Smith, 2005; Gregory, 2002; Šímová & Herben, 2012; Vinogradov & Anatskaya, 2006). The selection‐based theories of genome size evolution posit that selection acts on genome size indirectly through its effects on phenotypes (Cavalier‐Smith, 2005; Gregory, 2001). Accordingly, genome size may be shaped through its association with ecologically relevant traits, as genome streamlining or expansion could reflect adaptive shifts toward corresponding phenotypic optima (Hessen et al., 2010). However, our knowledge about genome size variation and its ecological consequences comes primarily from land plant and animal studies. Yet these organisms are complex multicellular entities in which the direct effects of genome size on individual cells may be buffered at the level of tissues or entire bodies. Moreover, any associations between genome size and phenotype can be further masked by somatic endopolyploidy, frequently occurring across multicellular taxa (Leitch & Dodsworth, 2017; Neiman et al., 2017). In contrast, unicellular eukaryotes (protists) provide more suitable system to investigate genome size variation and its phenotypic consequences, as a single cell constitutes the entire organism interacting directly with the environment. Except for vegetative cells, the entire body of a protist can be temporarily represented by a resting state by forming a cyst.

A large group of diverse protists producing cysts during their life cycles are chrysophytes (Chrysophyceae, Stramenopiles). This group of microalgae comprises of nearly 1600 species in 180 genera, with the genus *Mallomonas* representing the most species‐rich chrysophyte genera (Guiry & Guiry, 2026). The genus *Mallomonas* is characterized by producing species‐specific siliceous scales. Chrysophytes are also known as golden‐brown algae due to the color of the accessory pigment fucoxanthin (Jeffrey et al., 2011). They are an abundant, sometimes even a dominant, group of mainly freshwater planktonic protists (Bock et al., 2022; Kammerlander et al., 2015). Their morphology ranges from single‐celled (e.g., *Mallomonas*) or colonial flagellates (e.g., *Synura*) to branching forms (e.g., *Hydrurus*). Interestingly, most, if not all, species of chrysophyte produce a resting stage known as a stomatocyst or statospore (Sandgren, 1991). These cysts are siliceous, globular or ovoid structures typically 2–30 μm in diameter, bearing a species‐specific ornamented wall and a characteristic opening: the porus (Figure 1; Duff et al., 1995). The production of chrysophycean cysts occurs via sexual or asexual processes, regulated by both endogenous mechanisms and exogenous environmental triggers (Sandgren & Flanagin, 1986). After formation, cysts sink to the sediment where they remain as a long‐term seed bank, enabling population recovery once conditions improve (Ellegaard & Ribeiro, 2018). Stomatocysts also facilitate dispersal across aquatic habitats via wind, animals, or human‐mediated transport (Foissner, 2011). Thanks to robust silica walls, the stomatocysts are easily preserved in sediment and represent an important indicator of environmental conditions (Bai et al., 2021; Duff & Smol, 1995; Wilkinson, 1998). Consequently, chrysophytes provide a fossil record, otherwise rare among protists, which enables consideration of a number of ecological and evolutionary questions, while still leaving a lot unexplored. Only a small fraction of known stomatocyst morphotypes can be reliably linked to the chrysophyte species that produce them (Zeeb & Smol, 2001). Moreover, the mechanisms that shape cyst morphology are unclear. Given their functional significance and pronounced morphological variability, chrysophyte stomatocysts represent an intriguing trait through which to explore the potential phenotypic correlates of genome size.

**FIGURE 1:**
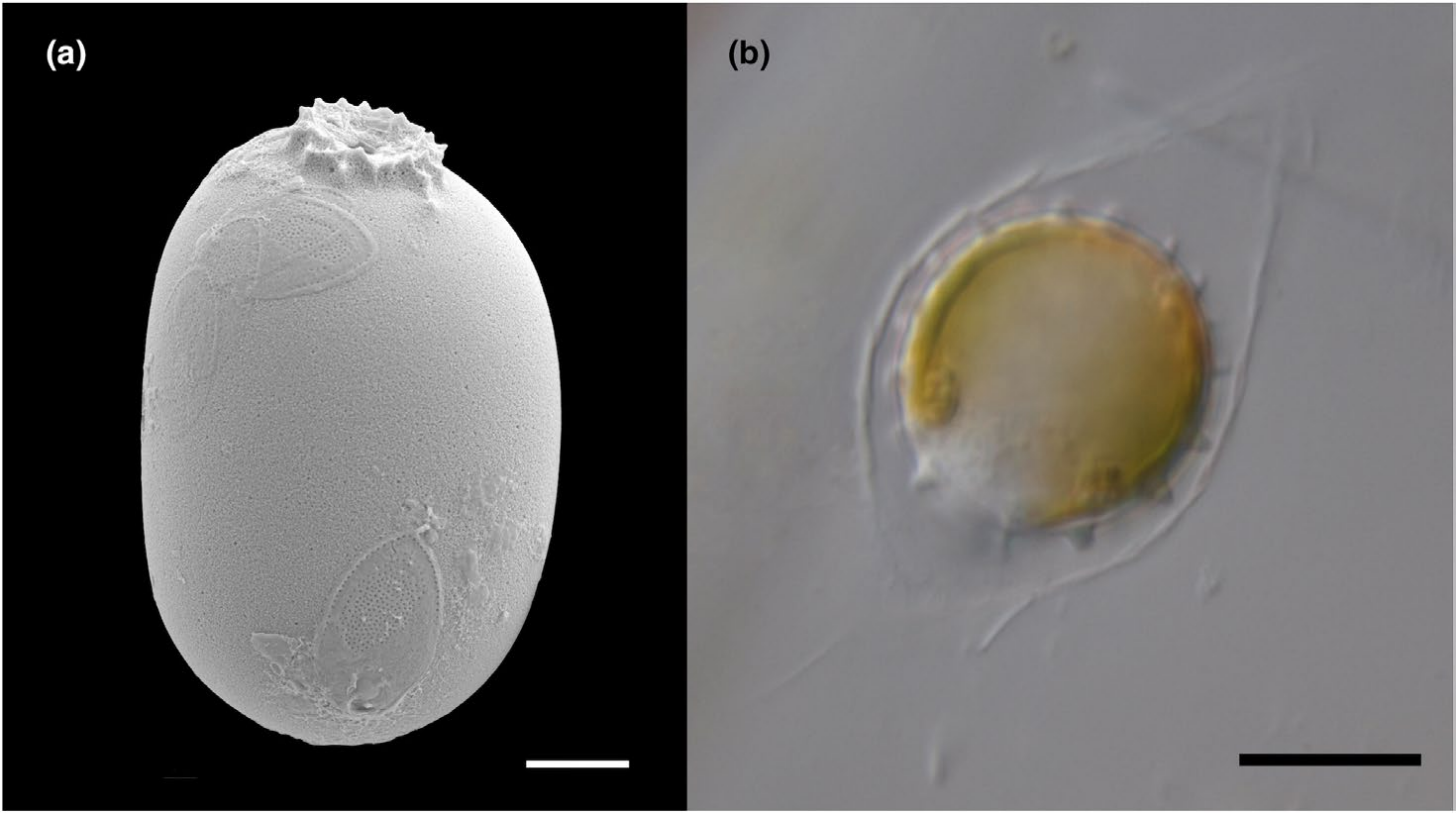
Variability in size of *Mallomonas* stomatocysts. (a) Small‐sized cyst of *M. akrokomos* (SEM) and (b) the largest known chrysophyte cyst of *M. caudata* surrounded by remnants of the original siliceous scale covering (LM). Scale bars: 2 μm (a) and 20 μm (b).

The aim of this study was to enhance our understanding of the ecological significance of protist genome size variation. By developing a database with genome size of chrysophycean genus *Mallomonas*, and by testing a link between genome size and available stomatocyst size data, we aimed to shed light on the phenotypic significance of genome size variation in this ecologically important lineage.

## MATERIALS AND METHODS

### Origin, cultivation, and species determination of new *Mallomonas* strains

New isolates of the genus *Mallomonas* were obtained from plankton samples collected from various water bodies mostly in the Czech Republic using a 20‐μm mesh plankton net. Water temperature, pH, and specific conductivity were obtained using a combined pH/conductometer (WTW 340i; WTW GmbH, Weilheim, Germany). The sampling details for strains used in this study are listed in Table S1. The single *Mallomonas* flagellates were captured by micro‐pipetting and transferred into separate culture wells filled with ~300 μL of modified liquid WC medium (original WC medium according to Guillard & Lorenzen, 1972, containing a double concentration of soluble silica source—Na_2_SiO_3_·9H_2_O—and with the addition of soil extract) buffered with TES. All cultures were maintained at 24°C with a constant light mode under illumination of 50 μmol photons · m^−2^ · s^−1^ (TLD 18 W/33 fluorescent lamps, Philips, Amsterdam, the Netherlands). Subsequently, the strains were transferred into Erlenmeyer flasks filled with 50 mL of growth medium and kept for longer cultivation with reinoculations into fresh medium every 3 months. To broaden our data set, we included additional *Mallomonas* strains from the study by Škaloud et al. (2025), from ongoing studies, and from the National Center for Marine Algae and Microbiota culture collection (*M*. *papillosa* CCMP476).

The species of the newly obtained *Mallomonas* strains were determined using an internal transcribed spacer region sequence of nuclear ribosomal DNA (ITS1, 5.8S, and ITS2 rDNA regions), except for a single strain (CA 03B). For this purpose, 90 mL of each culture was harvested by centrifugation and the superfluous material was removed by pipetting. Genomic DNA was extracted using 30 μL of InstaGene matrix (Bio‐Rad Laboratories, Hercules, California, United States), and the resulting supernatant was directly used as a polymerase chain reaction (PCR) template. The amplifications were performed using lineage‐specific primers KN1.1 (Wee et al., 2001) or Chryso_ITS_F and Chryso_ITS_R (Jadrná et al., 2021). The PCRs were carried out in a total volume of 10 μL with a PCR mix containing 0.1 μL of MyTaq DNA polymerase (Bioline, Memphis, Tenneesee, United States), 2 μL of MyTaq buffer (Bioline), 0.1 μL of each primer, 6.7 μL of double distilled water, and 1 μL of template DNA (not quantified). The amplifications were performed in Eppendorf Mastercycler ep Gradient 5341 (Eppendorf GmbH, Hamburg, Germany) using the following program: 1 min of denaturation at 95°C followed by 35 cycles of denaturation at 95°C (15 s), annealing at 52°C (30 s), and elongation at 72°C (40 s), and concluded with a final extension at 72°C (7 min) and held at 10°C. The PCR products were sized on a 0.8% agarose gel and then purified using ExoSAP‐IT™ PCR Product Cleanup Reagent (Applied Biosystems, Thermo Fisher Scientific, Waltham, Massachusetts, United States). The purified DNA templates were sequenced using the Sanger sequencing method at Macrogen, Inc. (Amsterdam, the Netherlands, https://www.macrogen‐europe.com). The obtained sequences were manually checked using SeqAssem ver. 9 (Hepperle, 2004) and identified using Basic Local Alignment Search Tool (BLAST) in the National Center for Biotechnology Information (NCBI) database and our personal ITS database.

The strain CA 03B was determined based on silica scale morphology using transmission electron microscopy (TEM). The sample was transferred onto Formvar‐coated copper grids, rinsed in four drops of distilled water, and air‐dried. Examination was performed using a TEM Jeol 1011 equipped with a Velvet CCD camera (Olympus Soft Imaging Solutions GmbH, Münster, Germany).

### Genome size estimation

To estimate genome size of newly isolated *Mallomonas* strains, we employed flow cytometry with propidium iodide staining (PI FCM). Approximately 3 weeks prior to analysis, cultures were transferred into fresh medium to ensure optimal growth. For each sample, 2 mL of culture in its exponential phase of growth was collected, centrifuged (5 min, 2040 × *g*; Eppendorf), and the supernatant was removed by pipetting. The pellet was then resuspended in 350 μL of ice‐cold nuclei isolation buffer Otto I (0.1 M citric acid, 0.5% Tween 20; Otto, 1990), causing an osmotic rupture of the cells and release of the sample nuclei. The suspension was mixed thoroughly and kept on ice. Depending on the estimated genome size of the strain, one of three plant species was selected as a (pseudo‐)internal reference standard: *Carex acutiformis*, wild clone (2C = 0.82 pg; Lipnerová et al., 2013), *Pisum sativum* cv. Ctirad (2C = 8.018 pg; Šmarda et al., 2014), or *Solanum pseudocapsicum*, commercial clone (2C = 2.59 pg; Temsch et al., 2010). For each standard, ~20 mg of fresh leaf tissue was chopped in 250 μL of ice‐cold Otto I buffer using a razor blade in a plastic Petri dish. The resulting suspension of standard nuclei was then mixed with the algal sample nuclei and together filtered through a 42‐μm nylon mesh into a 3.5‐mL cuvette for cytometric analysis. Following a 20‐min incubation at room temperature, 1 mL of staining solution consisting of Otto II buffer (0.4 M Na_2_HPO_4_·12H_2_O; Otto, 1990), 50 μg · mL^−1^ PI, 50 μg · mL^−1^ RNase IIA, and 2 μL · mL^−1^ β‐mercaptoethanol were added. The stained sample was immediately analyzed using a Partec CyFlow SL cytometer (Partec GmbH, Münster, Germany) equipped with a green solid‐state laser (Cobolt Samba, 532 nm, 100 mW). In each sample analysis, 5000 particles were measured and the resulting flow cyometry (FCM) histograms were analyzed using FloMax ver. 2.4d (Partec). The first sample peak in the FCM histogram was identified as G_1_ (vegetative nuclei), and a second peak with twice the relative fluorescence (if visible) as G_2_ (dividing nuclei). The absolute nuclear DNA content (C‐value) was calculated as sample G_1_ peak mean fluorescence/standard G_1_ peak mean fluorescence × standard 2C DNA content (according to Doležel, 2005). To minimize the effect of random instrumental shift, each strain was analyzed at least three times on separate days and the estimates averaged. Final genome size estimates are reported in absolute units per cell (pg of DNA and equivalent values in Mbp).

### Estimation of cyst volume and its relationship to genome size

We summarized the published dimensions of cysts and calculated their medium volume, either as a sphere or an ellipsoid, based on the shape of the cyst. The extension of the pore and the ornamentation on the cyst's surface were disregarded in our calculations due to a lack of data on these dimensions. Further, we summarized the published genome size data for chrysophytes, added the new data for the *Mallomonas* strains analyzed in this study, and calculated the median genome size value for each species. To test whether the variation in cyst size could be linked to variation in genome size, we applied a linear regression model in R v4.4.2 (R Core Team, 2024). Prior to this analysis, both variables were logarithmically transformed to improve their normality. The significance of the regression model was evaluated using an *F*‐test, and the significance of the regression coefficient was assessed using a *t*‐test.

## RESULTS

In this study, we obtained and established 61 new strains of *Mallomonas*. Strains were identified as 27 species of the genus *Mallomonas* using the *Synurales* nuclear ITS rDNA (nu ITS rDNA) reference database (Škaloud et al., 2025), except for strain CA 03B, which was determined based on silica scale morphology (Table S1). The absolute nuclear DNA amount was successfully determined for all strains included in this study (with addition of *Mallomonas* strains from previous studies, 85 strains in total, representing 31 *Mallomonas* species; Table S2). Flow cytometric measurements were precise, resulting in clearly delimited peaks in FCM histograms, with the average coefficient of variation among repeated estimates of 3.0%. The genome sizes of the analyzed *Mallomonas* strains varied widely, ranging from 0.15 pg (~143 Mbp) in *M*. *heterospina* to 11.25 pg (~11 Gbp) in *M*. *caudata*. Most species exhibited intraspecific genome size variability, which was particularly pronounced in *M*. *adamas* (0.33–0.99 pg), *M*. *annulata* (0.41–1.94 pg), *M*. *crassisquama* (0.83–3.15 pg), *M*. *oviformis* (1.19–3.68 pg), *M*. *papillosa* (0.37–0.83 pg), and *M*. *striata* var. *striata* (1.84–4.47 pg).

To analyze the relationship between genome size and cyst size in chrysophytes, we summarized data from the literature and supplemented them with new estimates obtained in this study, resulting in a data set for 54 chrysophyte species (Table 1). The smallest genome and cyst size were both in *Segregatospumella dracosaxi* (0.05 pg and 49 μm^3^, respectively), whereas the largest genome and cyst size occurred in *Mallomonas caudata* (12.4 pg and 14,130 μm^3^, respectively; Grossmann et al., 2016; Krieger, 1930; Olefeld et al., 2018). Genome size was significantly positively correlated with cyst size (*F*_1,52_ = 89.6, *p <* 0.001, $R_{\mathrm{adj}}^{2}$ = 0.626), indicating that an increase in genome size is associated with larger cyst volume in chrysophytes (Figure 2).

**TABLE 1:** Summary of published and new data on median genome size values of chrysophyte species, including standard deviations and median cyst volumes from the literature.

Taxon	Genome size		No. of taxa	Original reference	Cyst volume (μm^3^)	Original reference
	pg	Mbp				
*Cornospumella fuschlensis*	0.15	143	1	Olefeld et al. (2018)	58	Grossmann et al. (2016)
*Dinobryon bavaricum*	0.32	317	1	Olefeld et al. (2018)	697	Philipose (1953), Duff et al. (1995), and Snit'ko and Snit'ko (2024)
*Dinobryon divergens*	0.32	316 ± 21	4	Olefeld et al. (2018)	733	Zeeb and Smol (1993), Piątek (2007), Bazhenova and Kapustin (2021), and Snit'ko and Snit'ko (2024)
*Dinobryon pediforme*	0.23	222	1	Olefeld et al. (2018)	555	Piątek et al. (2020)
*Dinobryon sociale* var. *americana*	0.36	354	1	Olefeld et al. (2018)	547	Duff and Smol (1991)
*Chrysosphaerella longispina*	0.24	233 ± 39	3	Pusztai et al. (2026)	452	van de Vijver and Beyens (1997), Piątek and Piątek (2005), Piątek (2007), and Pang and Wang (2014)
*Mallomonas acaroides*	2.44	2389 ± 36	3	This study	4517	Conrad (1933) and Kristiansen and Preisig (2007)
*Mallomonas acaroides* var. *maramuresensis*	1.75	1710 ± 13	2	This study	1261	Peterfi and Momeu (1976) and Kristiansen and Preisig (2007)
*Mallomonas adamas*	0.66	646 ± 323	2	This study	1692	Kristiansen and Preisig (2007)
*Mallomonas akrokomos*	0.25	245 ± 42	8	This study	289	Conrad (1933), Péterfi (1966), Cronberg (1978), Duff et al. (1995), Cabala and Piatek (2004), Kristiansen and Preisig (2007), and Pang and Wang (2014)
*Mallomonas alpina*	1.29	1259 ± 292	4	This study	1354	Kristiansen and Preisig (2007)
*Mallomonas annulata*	0.68	677 ± 462	7	Olefeld et al. (2018); this study	697	Harris (1967) and Kristiansen and Preisig (2007)
*Mallomonas areolata*	3.51	3428 ± 195	2	This study	1189	Momeu and Péterfi (1983) and Kristiansen and Preisig (2007)
*Mallomonas caudata*	16.62	16,258 ± 6214	4	Olefeld et al. (2018); this study	9739	Krieger (1930), Conrad (1933), Asmund (1955), Cronberg (1988), and Duff et al. (1995)
*Mallomonas crassisquama*	1.74	1701 ± 711	6	This study	1324	Snit'ko and Snit'ko (2024)
*Mallomonas cratis*	3.62	3539	1	This study	3025	Peterfi and Momeu (1976) and Kristiansen and Preisig (2007)
*Mallomonas doignonii*	0.59	574 ± 4	3	This study	583	Ignatenko et al. (2023)
*Mallomonas elevata*	0.19	183	1	This study	510	Kim et al. (2014)
*Mallomonas eoa*	0.62	609 ± 22	2	This study	476	Cronberg (1973, 1978), Kristiansen and Preisig (2007), Pang and Wang (2014), and Ignatenko and Yatsenko‐Stepanova (2022)
*Mallomonas heterospina*	0.15	143	1	This study	2495	Cronberg (1989) and Kristiansen and Preisig (2007)
*Mallomonas insignis*	4.12	4034	1	This study	3372	Péterfi and Momeu (1977) and Kristiansen and Preisig (2007)
*Mallomonas intermedia*	1.05	1025 ± 53	3	This study	2295	Skogstad (1984) and Kristiansen and Preisig (2007)
*Mallomonas koreana*	0.50	488 ± 7	2	This study	449	Han and Jin (2008)
*Mallomonas lychenensis*	3.03	2961	1	This study	1908	Kristiansen and Preisig (2007)
*Mallomonas matvienkoae*	4.99	4883	1	This study	3880	Kristiansen and Preisig (2007)
*Mallomonas multiunca*	0.83	807 ± 144	2	This study	2320	Harris and Bradley (1960) and Kristiansen and Preisig (2007)
*Mallomonas ouradion*	0.45	437	1	This study	2805	Kalina (1969) and Kristiansen and Preisig (2007)
*Mallomonas oviformis*	2.54	2484 ± 1143	4	This study	3391	Kristiansen and Preisig (2007)
*Mallomonas papillosa*	0.47	459 ± 143	6	This study	651	Harris (1967), Belcher (1969), and Kristiansen and Preisig (2007)
*Mallomonas pumilio*	0.38	370 ± 66	3	This study	449	Asmund et al. (1982) and Kristiansen and Preisig (2007)
*Mallomonas silvicola*	0.40	396 ± 5	2	This study	1766	Asmund et al. (1982)
*Mallomonas schwemmlei*	1.07	1045 ± 47	2	This study	904	Kristiansen and Preisig (2007)
*Mallomonas striata* var. *serrata*	1.92	1879 ± 32	2	This study	756	Kristiansen and Preisig (2007)
*Mallomonas striata* var. *striata*	2.59	2533 ± 1050	5	This study	3052	Kristiansen and Preisig (2007)
*Mallomonas tonsurata*	0.86	840 ± 34	3	This study	1097	Kristiansen and Preisig (2007)
*Mallomonas torquata*	0.50	492	1	This study	606	Cronberg (1978) and Kristiansen and Preisig (2007)
*Mallomonas transsylvanica*	2.33	2282 ± 346	3	This study	2351	Kristiansen and Preisig (2007)
*Neotessella lapponica*	1.70	1662	1	Pusztai et al. (2026)	8678	Kristiansen and Preisig (2007)
*Ochromonas tuberculata*	0.81	793	1	Čertnerová et al. (2022)	1802	Mušálková (2021)
*Pedospumella encystans*	0.16	159 ± 24	2	Olefeld et al. (2018)	335	Findenig et al. (2010)
*Poteriospumella lacustris*	0.16	156	1	Olefeld et al. (2018)	58	Findenig et al. (2010)
*Segregatospumella dracosaxi*	0.09	88	1	Olefeld et al. (2018)	49	Grossmann et al. (2016)
*Spumella rivalis*	0.10	98	1	Olefeld et al. (2018)	70	Findenig et al. (2010) and Jeong et al. (2021)
*Spumella vulgaris*	0.30	294	1	Olefeld et al. (2018)	254	Findenig et al. (2010) and Jeong et al. (2021)
*Synura echinulata*	0.28	274 ± 48	6	Čertnerová et al. (2025)	1436	Starmach (1968) and Kristiansen and Preisig (2007)
*Synura petersenii*	1.19	1165 ± 256	131	Čertnerová and Škaloud (2020) and Škaloud et al. (2024)	1389	Starmach (1968), Klaveness and Guillard (1975), Kristiansen and Preisig (2007), Pang and Wang (2014), and Mušálková (2021)
*Synura sphagnicola*	0.39	385 ± 110	10	Olefeld et al. (2018) and Čertnerová et al. (2022)	1766	Kristiansen and Preisig (2007)
*Synura spinosa*	1.73	1687 ± 484	2	Čertnerová et al. (2025)	1436	Takahashi (1967)
*Synura splendida*	1.11	1086	1	Čertnerová et al. (2025)	5195	Kristiansen and Preisig (2007)
*Synura uvella*	0.80	782	1	Čertnerová et al. (2025)	1845	Starmach (1968) and Mušálková (2021)
*Uroglenopsis turfosa*	0.25	246	1	Pusztai et al. (2026)	523	Pusztai and Škaloud (2022)
*Urostipulosphaera lindiae*	0.19	182	1	Pusztai et al. (2026)	1217	Pusztai and Škaloud (2022)
*Urostipulosphaera articulata*	0.31	303	1	Pusztai et al. (2026)	1324	Pusztai and Škaloud (2022)
*Urostipulosphaera granulata*	0.22	213 ± 5	1	Pusztai et al. (2026)	485	Pusztai and Škaloud (2022) and An et al. (2025)

**FIGURE 2:**
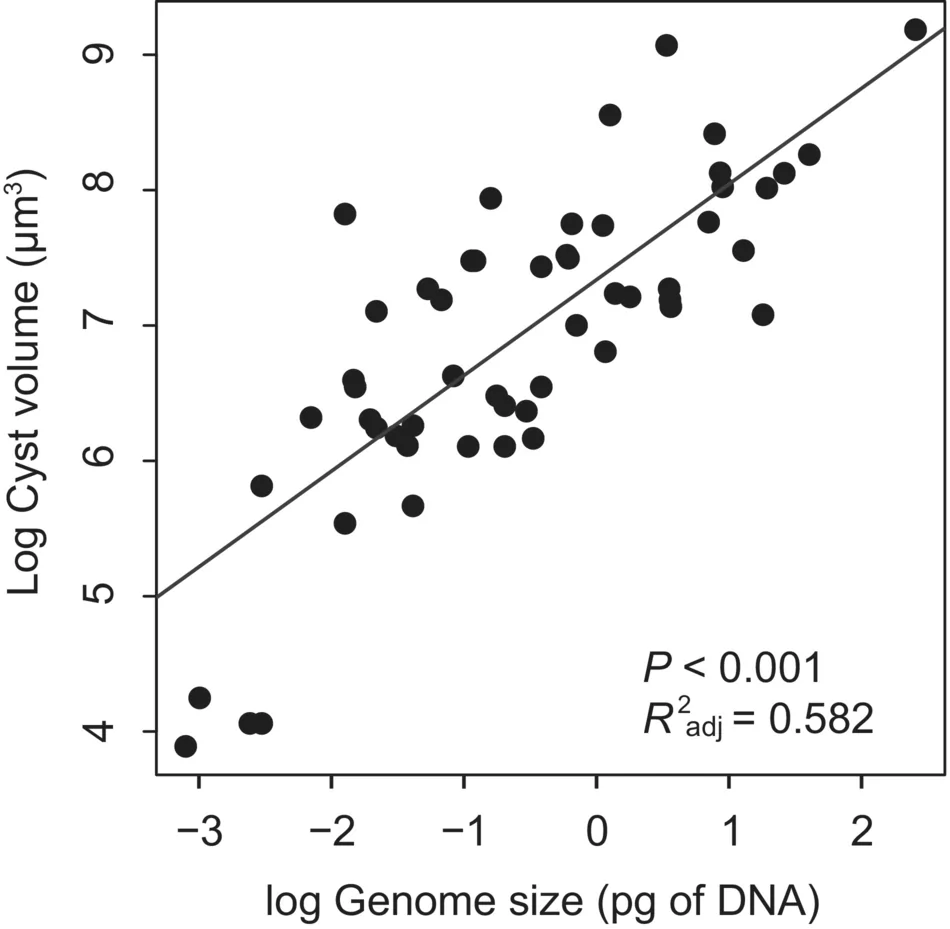
Linear regression model showing the relationship between log‐transformed genome size and cyst volume in chrysophytes.

## DISCUSSION

### Genome size and ploidy variation in the genus *Mallomonas*

Even though genome size in chrysophytes has recently become the focus of several studies (Čertnerová et al., 2022, 2025; Olefeld et al., 2018), the genus *Mallomonas* has so far remained largely overlooked. This is despite *Mallomonas* representing the most species‐rich genus among chrysophytes (Guiry & Guiry, 2026). Until now, genome size data had only been available for four *Mallomonas* species (Olefeld et al., 2018). In this study, we have provided 85 new genome size records for the genus *Mallomonas*, substantially broadening the available data set and deepening our knowledge about genome size variation within this genus. Our analyses revealed more than a 75‐fold difference in genome size among the examined strains, highlighting a great variability within the *Mallomonas* genus. Yet these additional data did not extend the existing genome size range for the whole chrysophytes (spanning from 0.05 pg in *Segregatospumella dracosaxi* to nearly 12.4 pg in *M. caudata*; Olefeld et al., 2018). A majority of analyzed *Mallomonas* species exhibited pronounced intraspecific genome size variation (Table S2). In some taxa (e.g., *M*. *annulata*, *M*. *pumilio*, and *M*. *transylvanica*), this variation appears gradual. Such continuous shifts in genome size could, in general, result from proliferation of transposable elements, nonhomologous recombination or segmental duplications (Devos et al., 2002; Lee & Kim, 2014; Stelzer et al., 2019). The intraspecific genome size variation and its phenotypic consequences were studied in detail in the species *Synura petersenii* (see Čertnerová & Škaloud, 2020), but the precise evolutionary mechanisms driving gradual genome size changes in chrysophytes remain unknown. In contrast, other species (e.g., *M*. *adamas*, *M*. *crassisquama*, *M*. *oviformis*, *M*. *papillosa*, *or M*. *striata* var. *striata*), displayed more pronounced, rather discrete differences in DNA content between strains, suggesting the presence of different ploidy levels. Recently, it has been revealed that chrysophytes have isomorphic haploid–diploid life cycles (Čertnerová et al., 2022), but in many cases, the observed variation here cannot be explained solely by different life‐cycle stages in different strains. Firstly, the DNA content among strains does not often follow exact multiples, implying combinations of ploidy‐level shifts and smaller‐scale genomic changes. Secondly, within some species, the DNA content variability seemed to correspond to different ploidy levels than the haploid and diploid level. For instance, the DNA content among *M*. *crassisquama* strains (0.83, 1.75, and 3.15 pg) or *M*. *annulata* (0.41, 0.76, and 1.94 pg) corresponded to combinations of three ploidy levels and intraspecific genome size variation, potentially altered by varying degrees of evolutionary genome downsizing. Similarly, *M*. *adamas* strains with genome sizes of 0.33 and 0.99 pg likely represent haploids and triploids, whereas *M*. *alpina* strains (1.0 and 1.5 pg) may correspond to diploids and triploids. The presence of triploids in chrysophytes has been previously detected by using comparative genomics (Majda et al., 2021), which revealed not only diploid and tetraploid but also many triploid individuals across several species. The frequent occurrence of triploid individuals in chrysophytes suggests hybridization between cytotypes and/or that unreduced gametes are often formed, highlighting a pattern of ploidy evolution in this group. Collectively, our data, together with those of Majda et al. (2021) and Čertnerová et al. (2022), suggest frequent whole‐genome duplications (polyploidization events) within chrysophytes. However, direct cytologic evidence of polyploidy (e.g., chromosome counts) remains lacking for chrysophytes, as is common among protists due to methodological challenges.

### Genome size—cyst size correlation

In this study, we reveal a significant positive correlation between genome size and cyst size across chrysophytes. To our knowledge, this is the first study to investigate such a relationship in any group of organisms. However, the relationships between genome size and other propagule size have been previously shown among eukaryotes, such as egg size in rotifers, shrimps, and fishes (Hardie & Hebert, 2004; Hultgren et al., 2021; Stelzer et al., 2021), pollen size in sweet potato (Srisuwan et al., 2019), spore size in ferns (Dyer et al., 2013), and seed mass in pines and Opuntioideae (Grotkopp et al., 2004; Lopes et al., 2021). The associations between genome size and propagule size were mostly positive, as in our study (but see, e.g., Beaulieu et al., 2007). The positive correlation between genome size and propagule size likely reflects underlying constraints related to cell size, which is, in general, strongly associated with genome size (Cavalier‐Smith, 2005). Although the genome size–cell size correlation is well documented and almost universal across eukaryotes, it seems weaker in chrysophytes (e.g., only 29% of variance explained across the chrysophyte genus *Synura*; Čertnerová et al., 2025). One of the explanations suggested is the lack of solid cell wall allowing greater flexibility in cell size (Čertnerová et al., 2025). In contrast, the relationship between genome size and cyst size appears much stronger, explaining over 60% of variation across chrysophytes (this study). This likely reflects the solid, silica‐based nature of stomatocysts. Therefore, cysts may serve as more reliable indicators of the link between genome size and organism (protist) size and be more useful for studying potential consequences.

Even though the observed genome size–cyst size correlation is strong, there are other influencing factors. Cyst morphology is understudied and remains unknown for most of the chrysophyte species. For example, it is presumed that cysts may arise from either the sexual or asexual phase of the life cycle (Sandgren, 1991; Skuja, 1950), and their morphology (but also size) might depend on their developmental origin. It has also been shown that environmental conditions during the time of their formation, such as temperature, pH, and light intensity, can slightly modify the morphology of chrysophyte silica scales (Nemcova et al., 2010; Němcová & Pichrtová, 2012; Pichrtová & Němcová, 2011), and similar effects are expected to influence the morphology of cysts as well. In addition, the vast majority of cyst size data are derived from electron microscopy, except for a few measurements done based on light microscopy (e.g., *Mallomonas caudata*; Conrad, 1933; Krieger, 1930). Since electron microscopy uses anhydrous samples, this may yield slightly different measurements (e.g., increase in cyst dimensions due to the presence of mucilaginous layers when using light microscopy, etc.).

Interestingly, species with relatively large cysts but small genomes (e.g., *Mallomonas heterospina*, *Urostipulosphaera lindiae*) occur more frequently than the opposite case. This pattern suggests that genome size may set a lower limit on cyst or cell size but does not necessarily enforce a comparably strict upper limit. The species with smaller genomes may produce larger cysts because of natural selection for increased storage capacity. Alternatively, larger cysts could arise from cells at a higher ploidy level, whereas genome size in the same species may have been measured from cultures maintained at a lower ploidy level.

Consequently, cyst size may affect ecological traits such as sedimentation rate or dispersal, analogous to size‐dependent dispersal in anemochorous plant seeds (Debain et al., 2003). In colonial chrysophytes (e.g., *Synura*), the cysts are developing directly in the colony, and larger cysts might impose a mechanical or energetic burden on the colony, potentially affecting their movement.

The strong positive correlation between genome size and cyst size provides a promising tool for estimating ancient genome sizes from fossil stomatocyst records. Interestingly, the same cannot be applied to silica scales, as their size shows no consistent relationship with genome size (Čertnerová et al., 2025).

The question remains as to whether the positive correlation between genome size and cyst size is a universal phenomenon among protists. If this association reflects regulation of the sizes of genomes, cells, and cysts, then the same pattern may be expected across other protist lineages. Therefore, examining genome and cyst sizes in other cyst‐forming unicellular organisms is essential to evaluate the broader evolutionary and morphological significance of this pattern.

## AUTHOR CONTRIBUTIONS

**Natálie Protić:** Data curation (equal); investigation (lead); writing – review and editing (supporting). **Iva Jadrná:** Investigation (equal); methodology (equal); resources (equal); writing – review and editing (supporting). **Pavel Škaloud:** Data curation (equal); funding acquisition (equal); investigation (equal); methodology (equal); writing – review and editing (equal). **Petr Knotek:** Investigation (supporting); methodology (supporting); resources (equal); visualization (equal); writing – review and editing (supporting). **Dora Čertnerová:** Conceptualization (lead); data curation (equal); formal analysis (lead); funding acquisition (lead); investigation (supporting); methodology (lead); project administration (lead); supervision (lead); validation (lead); visualization (equal); writing – original draft (lead); writing – review and editing (equal).

## Supporting information


**Table S1.** Collection details for the *Mallomonas* strains analyzed for genome size, with listed GenBank accession numbers for the nuclear ITS rDNA region sequences. The new sequences are indicated in bold.

**Table S2.** Genome sizes of the *Mallomonas* strains analyzed in this study, including standard deviations (*SD*) and the plant reference standards.
