# Supplementary material for: Linking variation in genome size to cyst size in chrysophytes
Source: J Phycol. 2026 Aug 7;62(5):1671–82. doi: 10.1111/jpy.70217 (PMC13659389; doi:10.1111/jpy.70217)
Supplement: Supplementary file 1 [file JPY-62-1671-s001.docx]

Table S2. Genome sizes of the *Mallomonas* strains analyzed in this study, including standard deviations (*SD*) and the plant reference standards.

| Species | Strain | Mean sample/standard ratio of  relative fluorescence | Genome size | | FCM standard |
| --- | --- | --- | --- | --- | --- |
|  |  |  | [Mbp] | [pg] |  |
| *M*. *acaroides* | CZ 03A | 2.979 | 2389 ± 0.02 | 2.44 | *Carex acutiformis* |
|  | CZ 07J | 3.030 | 2430 ± 0.00 | 2.48 | *Carex acutiformis* |
|  | CZ 22A | 2.920 | 2342 ± 0.03 | 2.39 | *Carex acutiformis* |
| *M*. *actinoloma* var. *maramuresensis* | CZ 104D | 2.116 | 1697 ± 0.04 | 1.74 | *Carex acutiformis* |
|  | CZ 104E | 2.149 | 1724 ± 0.03 | 1.76 | *Carex acutiformis* |
| *M*. *adamas* | CZ 38K | 0.383 | 970 ± 0.01 | 0.99 | *Solanum pseudocapsicum* |
|  | CZ 40D | 0.403 | 323 ± 0.02 | 0.33 | *Carex acutiformis* |
| *M*. *akrokomos* | CZ 30G1 | 0.308 | 247 ± 0.00 | 0.25 | *Carex acutiformis* |
|  | CZ 31C | 0.312 | 250 ± 0.00 | 0.26 | *Carex acutiformis* |
|  | CZ 34L | 0.335 | 269 ± 0.01 | 0.27 | *Carex acutiformis* |
|  | CZ 35I | 0.314 | 252 ± 0.00 | 0.26 | *Carex acutiformis* |
|  | CZ 138S | 0.200 | 160 ± 0.01 | 0.16 | *Carex acutiformis* |
|  | CZ 145A | 0.214 | 172 ± 0.00 | 0.18 | *Carex acutiformis* |
|  | CZ 183A | 0.213 | 171 ± 0.00 | 0.17 | *Carex acutiformis* |
|  | NL 13D | 0.303 | 243 ± 0.00 | 0.25 | *Carex acutiformis* |
| *M*. *alpina* | CZ 22D | 1.882 | 1509 ± 0.03 | 1.54 | *Carex acutiformis* |
|  | CZ 23D | 1.964 | 1575 ± 0.08 | 1.61 | *Carex acutiformis* |
|  | CZ 35A | 1.258 | 1009 ± 0.02 | 1.03 | *Carex acutiformis* |
|  | CZ 35G | 1.146 | 919 ± 0.01 | 0.94 | *Carex acutiformis* |
| *M*. *annulata* | CZ 19A | 0.248 | 629 ± 0.03 | 0.64 | *Solanum pseudocapsicum* |
|  | CZ 23C | 0.199 | 503 ± 0.00 | 0.51 | *Solanum pseudocapsicum* |
|  | CZ 23E | 0.747 | 1893 ± 0.02 | 1.94 | *Solanum pseudocapsicum* |
|  | CZ 34K | 0.290 | 734 ± 0.00 | 0.75 | *Solanum pseudocapsicum* |
|  | CZ 207I | 0.292 | 741 ± 0.00 | 0.76 | *Solanum pseudocapsicum* |
|  | CZ 212AZ | 0.264 | 667 ± 0.00 | 0.68 | *Solanum pseudocapsicum* |
|  | CZ 212BD | 0.159 | 404 ± 0.00 | 0.41 | *Solanum pseudocapsicum* |
| *M*. *areolata* | CZ 35M | 1.431 | 3623 ± 0.03 | 3.70 | *Solanum pseudocapsicum* |
|  | CZ 138K | 1.277 | 3233 ± 0.09 | 3.31 | *Solanum pseudocapsicum* |
| *M*. *caudata* | CZ 25J | 1.343 | 10298 ± 0.28 | 10.53 | *Pisum sativum* |
|  | CZ 92A | 1.435 | 11002 ± 0.27 | 11.25 | *Pisum sativum* |
| *M*. *crassisquama* | CZ 04A | 0.669 | 1695 ± 0.00 | 1.73 | *Solanum pseudocapsicum* |
|  | CZ 07G | 0.674 | 1708 ± 0.01 | 1.75 | *Solanum pseudocapsicum* |
|  | CZ 47F | 0.322 | 815 ± 0.00 | 0.83 | *Solanum pseudocapsicum* |
|  | CZ 53B | 1.217 | 3081 ± 0.03 | 3.15 | *Solanum pseudocapsicum* |
|  | NO 01F | 0.484 | 1226 ± 0.02 | 1.25 | *Solanum pseudocapsicum* |
|  | SW 62C | 2.588 | 2075 ± 0.00 | 2.12 | *Carex acutiformis* |
| *M*. *cratis* | CZ 18J | 1.397 | 3539 ± 0.02 | 3.62 | *Solanum pseudocapsicum* |
| *M*. *doignonii* | CZ 08N | 0.225 | 571 ± 0.01 | 0.58 | *Solanum pseudocapsicum* |
|  | NL 06A | 0.228 | 578 ± 0.00 | 0.59 | *Solanum pseudocapsicum* |
| *M*. *elevata* | CZ 154M | 0.228 | 183 ± 0.00 | 0.19 | *Carex acutiformis* |
| *M*. *eoa* | CZ 115B | 0.232 | 587 ± 0.02 | 0.60 | *Solanum pseudocapsicum* |
|  | CZ 115D | 0.249 | 632 ± 0.02 | 0.65 | *Solanum pseudocapsicum* |
| *M*. *heterospina* | CZ 08B | 0.178 | 143 ± 0.00 | 0.15 | *Carex acutiformis* |
| *M*. *insignis* | CZ 162H | 0.526 | 4034 ± 0.04 | 4.12 | *Pisum sativum* |
| *M*. *intermedia* | CZ 34M | 0.446 | 1129 ± 0.02 | 1.15 | *Solanum pseudocapsicum* |
|  | CZ 35C | 0.399 | 1009 ± 0.01 | 1.03 | *Solanum pseudocapsicum* |
|  | CZ 110B | 0.405 | 1025 ± 0.02 | 1.05 | *Solanum pseudocapsicum* |
| *M*. *koreana* | CZ 35AD | 0.601 | 482 ± 0.02 | 0.49 | *Carex acutiformis* |
|  | CZ 83E | 0.617 | 495 ± 0.00 | 0.51 | *Carex acutiformis* |
| *M*. *lychenensis* | HU 02F | 3.692 | 2961 ± 0.04 | 3.03 | *Carex acutiformis* |
| *M*. *matvienkoae* | CZ 153I | 1.948 | 4883 ± 0.08 | 4.99 | *Solanum pseudocapsicum* |
| *M*. *multiunca* | CZ 31E | 0.376 | 951 ± 0.01 | 0.97 | *Solanum pseudocapsicum* |
|  | NL 42C | 0.262 | 664 ± 0.02 | 0.68 | *Solanum pseudocapsicum* |
| *M*. *ouradion* | CZ 159D | 0.545 | 437 ± 0.01 | 0.45 | *Carex acutiformis* |
| *M*. *oviformis* | CZ 93H | 1.258 | 3544 ± 0.04 | 3.62 | *Solanum pseudocapsicum* |
|  | CZ 99D | 1.422 | 3601 ± 0.04 | 3.68 | *Solanum pseudocapsicum* |
|  | CZ 120F | 0.562 | 1424 ± 0.02 | 1.46 | *Solanum pseudocapsicum* |
|  | CZ 216K | 1.450 | 1163 ± 0.00 | 1.19 | *Carex acutiformis* |
| *M*. *papillosa* | CCMP 476 | 0.646 | 518 ± 0.01 | 0.53 | *Carex acutiformis* |
|  | CZ 03F | 0.319 | 808 ± 0.02 | 0.83 | *Solanum pseudocapsicum* |
|  | CZ 04C | 0.526 | 422 ± 0.01 | 0.43 | *Carex acutiformis* |
|  | CZ 34J | 0.564 | 453 ± 0.01 | 0.46 | *Carex acutiformis* |
|  | CZ 38E | 0.452 | 362 ± 0.01 | 0.37 | *Carex acutiformis* |
|  | NL 18B | 0.581 | 466 ± 0.01 | 0.48 | *Carex acutiformis* |
| *M*. cf. *pumilio* | CZ 114E | 0.461 | 370 ± 0.00 | 0.38 | *Carex acutiformis* |
|  | CZ 212AA | 0.501 | 401 ± 0.00 | 0.41 | *Carex acutiformis* |
|  | CZ 212AH | 0.308 | 247 ± 0.00 | 0.25 | *Carex acutiformis* |
| *M*. *schwemmlei* | CZ 33F | 0.431 | 1092 ± 0.01 | 1.12 | *Solanum pseudocapsicum* |
|  | CZ 33J | 0.394 | 998 ± 0.01 | 1.02 | *Solanum pseudocapsicum* |
| *M*. *silvicola* | CZ 157C | 0.500 | 401 ± 0.01 | 0.41 | *Carex acutiformis* |
|  | CZ 161C | 0.487 | 391 ± 0.00 | 0.40 | *Carex acutiformis* |
| *M*. *striata* var. *serrata* | CA 03B | 0.412 | 331 ± 0.00 | 0.34 | *Carex acutiformis* |
|  | US 29B | 0.426 | 342 ± 0.01 | 0.35 | *Carex acutiformis* |
| *M*. *striata* var. *striata* | CZ 04D | 2.240 | 1796 ± 0.00 | 1.84 | *Carex acutiformis* |
|  | CZ 12D | 2.640 | 2117 ± 0.01 | 2.16 | *Carex acutiformis* |
|  | CZ 33H | 3.158 | 2533 ± 0.02 | 2.59 | *Carex acutiformis* |
|  | CZ 71B | 0.571 | 4376 ± 0.07 | 4.47 | *Pisum sativum* |
|  | CZ 154D | 0.533 | 4085 ± 0.09 | 4.18 | *Pisum sativum* |
| *M*. *tonsurata* | CZ 09B | 0.359 | 909 ± 0.01 | 0.93 | *Solanum pseudocapsicum* |
|  | CZ 12A | 0.332 | 840 ± 0.01 | 0.86 | *Solanum pseudocapsicum* |
|  | CZ 32F | 0.329 | 833 ± 0.02 | 0.85 | *Solanum pseudocapsicum* |
| *M*. *torquata* | CZ 132E | 0.613 | 492 ± 0.01 | 0.50 | *Carex acutiformis* |
| *M*. *transsylvanica* | CZ 46F | 2.845 | 2282 ± 0.07 | 2.33 | *Carex acutiformis* |
|  | CZ 50B | 0.641 | 1624 ± 0.08 | 1.66 | *Solanum pseudocapsicum* |
|  | CZ 88A | 3.014 | 2417 ± 0.11 | 2.47 | *Carex acutiformis* |
