# Supplementary material for: Linking variation in genome size to cyst size in chrysophytes
Source: J Phycol. 2026 Aug 7;62(5):1671–82. doi: 10.1111/jpy.70217 (PMC13659389; doi:10.1111/jpy.70217)
Supplement: Supplementary file 2 [file JPY-62-1671-s002.docx]

Table S1. Collection details for the *Mallomonas* strains analyzed for genome size, with listed GenBank accession numbers for the nuclear ITS rDNA region sequences. The new sequences are indicated in bold.

| Species | Strain | Collection site | GPS coordinates | Sampling date | in situ measured environmental variables | | | nr ITS GenBank accession number |
| --- | --- | --- | --- | --- | --- | --- | --- | --- |
|  |  |  |  |  | Water temperature (°C) | pH | Conductivity (µS cm^-1^) |  |
| *M*. *acaroides* | CZ 03A | Czechia, Podhorni pond | 50.1423467, 13.8892633 | 13.02.2020 | 2.1 | 7.8 | 512 | **PZ067744** |
|  | CZ 07J | Czechia, Nová Ves nad Luznici, oxbow lake | 48.83514, 14.9271 | 12.03.2020 | 10.7 | 8.1 | 230 | **PZ067745** |
|  | CZ 22A | Czechia, Tuboz pond | 50.4905461, 14.5997953 | 12.04.2020 | 15 | 8.5 | 470 | **PZ067746** |
| *M*. *actinoloma* var. *maramuresensis* | CZ 104D | Czechia, pool Na Plachte | 50.1886000, 15.8607278 | 26.10.2020 | 12.1 | 7 | 51 | **PZ067747** |
|  | CZ 104E | Czechia, pool Na Plachte | 50.1886000, 15.8607278 | 26.10.2020 | 12.1 | 7 | 51 | **PZ067748** |
| *M*. *adamas* | CZ 38K | Czechia, Mariansky pond | 50.54551, 14.67669 | 04.05.2020 | 10.8 | 6.4 | 121 | **PZ067749** |
|  | CZ 40D | Czechia, Swamp peat bog | 50.5760483, 14.6696636 | 04.05.2020 | 12.8 | 6.2 | 79 | **PZ067750** |
| *M*. *akrokomos* | CZ 30G1 | Czechia, forest pond near Zasada | 50.6925339, 15.2629731 | 17.04.2020 | - | 6.5 | 98 | **PZ067751** |
|  | CZ 31C | Czechia, Dvory, alluvial pool | 48.85259, 14.9087 | 27.04.2020 | 13.6 | 6.9 | 238 | **PZ067752** |
|  | CZ 34L | Czechia, Suchdol, Luznice oxbow lake | 48.9088967, 14.8907903 | 27.04.2020 | 13.8 | 7.1 | 207 | **PZ067753** |
|  | CZ 35I | Czechia, Zlata stoka canal | 48.9518128, 14.8681525 | 27.04.2020 | 12.2 | 7.7 | 288 | **PZ067754** |
|  | CZ 138S | Czechia, Morava oxbow river | 48.8027053, 17.0940214 | 08.11.2020 | 9.3 | 7.3 | 372 | PV036614 |
|  | CZ 145A | Czechia, Pivovarsky pond | 50.7553817, 15.0921261 | 13.11.2020 | 7.1 | 7.5 | 460 | **PZ067755** |
|  | CZ 183A | Czechia, Mala piskovna | 48.8963006, 14.9077469 | 23.10.2021 | 11.1 | 7 | 159 | **PZ067756** |
|  | NL 13D | the Netherlands, Nederasselt, unnamed pool | 51.7712175, 5.7552169 | 13.11.2021 | 8.6 | 7.8 | 454 | **PZ067757** |
| *M*. *alpina* | CZ 22D | Czechia, Tuboz pond | 50.4905461, 14.5997953 | 12.04.2020 | 15 | 8.5 | 470 | **PZ067758** |
|  | CZ 23D | Czechia, Stribrnik pond | 50.4617539, 14.6048111 | 12.04.2020 | 14.9 | 8.4 | 418 | **PZ067759** |
|  | CZ 35A | Czechia, Zlata stoka canal | 48.9518128, 14.8681525 | 27.04.2020 | 12.2 | 7.7 | 288 | **PZ067760** |
|  | CZ 35G | Czechia, Zlata stoka canal | 48.9518128, 14.8681525 | 27.04.2020 | 12.2 | 7.7 | 288 | **PZ067761** |
| *M*. *annulata* | CZ 19A | Czechia, Krtiny pond | 50.0256500, 14.5350828 | 06.04.2020 | 12 | 7.6 | 1052 | PV036620 |
|  | CZ 23C | Czechia, Stribrnik pond | 50.4617539, 14.6048111 | 12.04.2020 | 14.9 | 8.4 | 418 | **PZ067762** |
|  | CZ 23E | Czechia, Stribrnik pond | 50.4617539, 14.6048111 | 12.04.2020 | 14.9 | 8.4 | 418 | **PZ067763** |
|  | CZ 34K | Czechia, Luznice oxbow lake, Suchdol | 48.9088967, 14.8907903 | 27.04.2020 | 13.8 | 7.1 | 207 | **PZ067764** |
|  | CZ 207I | Czechia, Vizir | 48.9644631, 14.8897156 | 16.01.2023 | 4.9 | 7.7 | 109 | **PZ067765** |
|  | CZ 212AZ | Czechia, peat bog lake Spalena Borkovna | 48.9810481, 14.7997919 | 16.01.2023 | 2.6 | 6.4 | 134 | **PZ067766** |
|  | CZ 212BD | Czechia, peat bog lake Spalena Borkovna | 48.9810481, 14.7997919 | 16.01.2023 | 2.6 | 6.4 | 134 | **PZ067767** |
| *M*. *areolata* | CZ 35M | Czechia, Zlata stoka canal | 48.9518128, 14.8681525 | 27.04.2020 | 12.2 | 7.7 | 288 | PV036621 |
|  | CZ 138K | Czechia, Morava oxbow river | 48.8027053, 17.0940214 | 08.11.2020 | 9.3 | 7.3 | 372 | **PZ067768** |
| *M*. *caudata* | CZ 25J | Czechia, Harasov lake | 50.4120428, 14.5752211 | 12.04.2020 | 14.5 | 8.5 | 388 | **PZ067769** |
|  | CZ 92A | Czechia, Kurfurstovo rameno oxbow lake | 49.6612536, 17.2125003 | 08.10.2020 | 12.5 | 7.3 | 698 | PV036624 |
| *M*. *crassisquama* | CZ 04A | Czechia, Malse river | 48.975711, 14.470721 | 24.02.2020 | 4 | 8.4 | 163 | PV036630 |
|  | CZ 07G | Czechia, Nova Ves nad Luznici, oxbow lake | 48.83514, 14.9271 | 12.03.2020 | 10.7 | 8.1 | 230 | **PZ067770** |
|  | CZ 47F | Czechia, Pod cestou pond | 49.5586269, 15.9045156 | 19.04.2020 | 15.2 | 7.7 | 96 | **PZ067771** |
|  | CZ 53B | Czechia, Male Darko pond | 49.6644739, 15.8800561 | 11.05.2020 | 17.7 | 8.14 | 150 | **PZ067772** |
|  | NO 01F | Norway, Dovrevegen, unnamed pond | 62.5484839, 9.6308597 | 29.08.2021 | - | 6.7 | 545 | **PZ067773** |
|  | SW 62C | Sweden, Stockholm, Bergianska tradgarden | 59.3680303, 18.0452797 | 20.10.2022 | - | - | - | **PZ067774** |
| *M*. *cratis* | CZ 18J | Czechia, Homolka pond | 50.0268994, 14.5452108 | 06.04.2020 | 13.6 | 8.3 | 743 | **PZ067775** |
| *M*. *doignonii* | CZ 08N | Czechia, Dvory, alluvial pool | 48.85259, 14.9087 | 12.03.2020 | 10.5 | 8.2 | 228 | **PZ067776** |
|  | NL 06A | The Netherlands, Oisterwijk, unnamed pond | 51.5709183, 5.2172714 | 12.11.2021 | 7.1 | 7.4 | 62 | **PZ067777** |
| *M*. *elevata* | CZ 154M | Czechia, Zlata stoka canal | 48.9848758, 14.7882978 | 11.12.2020 | 3.8 | 7.1 | 196 | **PZ067778** |
| *M*. *eoa* | CZ 115B | Czechia, Kladsky pond | 50.0277756, 12.6750450 | 31.10.2020 | 7.6 | 5.9 | 49 | PV036638 |
|  | CZ 115D | Czechia, Kladsky pond | 50.0277756, 12.6750450 | 31.10.2020 | 7.6 | 5.9 | 49 | **PZ067779** |
| *M*. *heterospina* | CZ 08B | Czechia, Dvory, alluvial pool | 48.85259, 14.9087 | 12.03.2020 | 10.5 | 8.2 | 228 | **PZ067780** |
| *M*. *insignis* | CZ 162H | Czechia, Spalena Borkovna peat bog | 48.9809317, 14.8000531 | 07.04.2021 | 7.5 | 6.8 | 320 | PV036646 |
| *M*. *intermedia* | CZ 34M | Czechia, Suchdol, Luznice oxbow lake | 48.9088967, 14.8907903 | 27.04.2020 | 13.8 | 7.1 | 207 | PV036647 |
|  | CZ 35C | Czechia, Zlata stoka canal | 48.9518128, 14.8681525 | 27.04.2020 | 12.2 | 7.7 | 288 | **PZ067781** |
|  | CZ 110B | Czechia, oxbow lake near Pekna | 48.8488272, 13.9246825 | 30.10.2020 | 7.5 | 6.7 | 79 | **PZ067782** |
| *M*. *koreana* | CZ 35AD | Czechia, Zlata stoka canal | 48.9518128, 14.8681525 | 27.04.2020 | 12.2 | 7.7 | 288 | PV036650 |
|  | CZ 83E | Czechia, Zlata stoka canal | 48.9681442, 14.8393619 | 09.10.2020 | 11.5 | 7.3 | 176 | **PZ067783** |
| *M*. *lychenensis* | HU 02F | Hungary, Risztics parlag | 47.7058658, 19.0426764 | 15.04.2022 | 10.8 | 6.8 | 173 | PV036655 |
| *M*. *matvienkoae* | CZ 153I | Czechia, Spalena Borkovna peat bog | 48.9804136, 14.8003006 | 11.12.2020 | 3.2 | 6.2 | 159 | **PZ067784** |
| *M*. *multiunca* | CZ 31E | Czechia, Dvory, alluvial pool | 48.85259, 14.9087 | 27.04.2020 | 13.6 | 6.9 | 238 | PV036659 |
|  | NL 42C | The Netherlands, Rolvennen lake | 51.1710200, 6.1068900 | 17.11.2021 | 6.6 | 6 | 46 | **PZ067785** |
| *M*. *ouradion* | CZ 159D | Czechia, forest pond Gabom | 49.0001461, 14.8128283 | 11.12.2020 | 3.8 | 5.2 | 98 | PV036660 |
| *M*. *oviformis* | CZ 99D | Czechia, Vyskyt pond | 50.1788511, 15.9144339 | 26.10.2020 | 11.1 | 7.8 | 532 | **PZ067786** |
|  | CZ 93H | Czechia, wetland, Horka nad Moravou | 49.6410397, 17.2019122 | 08.10.2020 | 13.2 | 7.5 | 515 | PV036661 |
|  | CZ 120F | Czechia, Farsky pond | 49.5586869, 15.9071714 | 06.11.2020 | 6.7 | 6.6 | 117 | **PZ067787** |
|  | CZ 216K | Czechia, Rod pond | 49.1190364, 14.7491533 | 18.01.2023 | 3.2 | 7.1 | 257 | **PZ067788** |
| *M*. *papillosa* | CCMP 476 | UK, England, a freshwater saltmarsh | - | - | - | - | - |  |
|  | CZ 03F | Czechia, Podhorni pond | 50.1423467, 13.8892633 | 13.02.2020 | 2.1 | 7.8 | 512 | **PZ067789** |
|  | CZ 04C | Czechia, Malse river | 48.975711, 14.470721 | 24.02.2020 | 4 | 8.4 | 163 | PV036666 |
|  | CZ 34J | Czechia, Luznice oxbow lake, Suchdol | 48.9088967, 14.8907903 | 27.04.2020 | 13.8 | 7.1 | 207 | **PZ067790** |
|  | CZ 38E | Czechia, Mariansky pond | 50.54551, 14.67669 | 04.05.2020 | 10.8 | 6.4 | 121 | **PZ067791** |
|  | NL 18B | The Netherlands, unnamed pond in Plasmolen | 51.7380808, 5.9300058 | 13.11.2021 | 10.3 | 7.6 | 352 | PV036667 |
| *M*. cf. *pumilio* | CZ 114E | Czechia, forest pond | 49.9800311, 12.7870547 | 31.10.2020 | 9 | 6.6 | 320 | PV036679 |
|  | CZ 212AA | Czechia, peat bog lake Spalena Borkovna | 48.9810481, 14.7997919 | 16.01.2023 | 2.6 | 6.4 | 134 | **PZ067792** |
|  | CZ 212AH | Czechia, peat bog lake Spalena Borkovna | 48.9810481, 14.7997919 | 16.01.2023 | 2.6 | 6.4 | 134 | **PZ067793** |
| *M*. *schwemmlei* | CZ 33F | Czechia, Dvory, Luznice oxbow lake | 48.8564053, 14.8993053 | 27.04.2020 | 13.2 | 7.8 | 130 | **PZ067794** |
|  | CZ 33J | Czechia, Dvory, Luznice oxbow lake | 48.8564053, 14.8993053 | 27.04.2020 | 13.2 | 7.8 | 130 | PV036683 |
| *M*. *silvicola* | CZ 157C | Czechia, forest pond Congo | 49.0003089, 14.8128069 | 11.12.2020 | 3.9 | 5.8 | 31 | PV036684 |
|  | CZ 161C | Czechia, Majdalena, forest pond | 48.9869442, 14.8332058 | 11.12.2020 | 3.3 | 5.9 | 22 | **PZ067795** |
| *M*. *striata* | CZ 04D | Czechia, Malse river | 48.975711, 14.470721 | 24.02.2020 | 4 | 8.4 | 163 | PV036685 |
|  | CZ 12D | Czechia, Dvory, Luznice alluvial pool | 48.86438, 14.89414 | 13.03.2020 | 10.2 | 7.9 | 179 | **PZ067796** |
|  | CZ 33H | Czechia, Dvory, Luznice oxbow lake | 48.8564053, 14.8993053 | 27.04.2020 | 13.2 | 7.8 | 130 | **PZ067797** |
|  | CZ 71B | Czechia, Svratka pond | 49.7069431, 16.0270547 | 18.05.2020 | 12.2 | 6.8 | 114 | PV036687 |
|  | CZ 154D | Czechia, Zlata stoka canal | 48.9848758, 14.7882978 | 11.12.2020 | 3.8 | 7.1 | 196 | **PZ067798** |
| *M*. *striata* var. *serrata* | CA 03B | Czechia, Podhorni pond | 50.1423467, 13.8892633 | 13.02.2020 | 2.1 | 7.8 | 512 | **-** |
|  | US 29B | The United States, Otis, unnamed lake | 42.2450994, -73.1546747 | 09.05.2022 | 16.7 | 6.5 | 31 | **PZ067799** |
| *M*. *tonsurata* | CZ 09B | Czechia, Dvory, Luznice oxbow lake | 48.85101, 14.90807 | 12.03.2020 | 8.5 | 8.7 | 210 | **PZ067800** |
|  | CZ 12A | Czechia, Dvory, Luznice oxbow lake | 48.86438, 14.89414 | 13.03.2020 | 10.2 | 7.9 | 179 | **PZ067801** |
|  | CZ 32F | Czechia, Dvory, Luznice oxbow lake | 48.86438, 14.89414 | 27.04.2020 | 13.8 | 6.8 | 169 | PV036692 |
| *M*. *torquata* | CZ 132E | Czechia, Lanzhot, Cahnov – Soutok, canal | 48.6628856, 16.9375286 | 08.11.2020 | 8.7 | 7.5 | 908 | PV036693 |
| *M*. *transsylvanica* | CZ 46F | Czechia, Velky Babin pond | 49.5422786, 15.8974917 | 20.04.2020 | 17.8 | 6.5 | 61 | PV036694 |
|  | CZ 50B | Czechia, Polnicka, Fejtovsky pond | 49.6180339, 15.9017161 | 21.04.2020 | 11.8 | 7.4 | 97 | **PZ067802** |
|  | CZ 88A | Czechia, Borkovicka blata, canal | 49.2355644, 14.6235697 | 09.10.2020 | 11.5 | 6.7 | 104 | **PZ067803** |
